# Bagaza Virus in Partridges and Pheasants, Spain, 2010

**DOI:** 10.3201/eid1708.110077

**Published:** 2011-08

**Authors:** Montserrat Agüero, Jovita Fernández-Pinero, Dolores Buitrago, Azucena Sánchez, Maia Elizalde, Elena San Miguel, Ruben Villalba, Francisco Llorente, Miguel Ángel Jiménez-Clavero

**Affiliations:** Author affiliations: Laboratorio Central de Veterinaria, Algete, Spain (M. Agüero, D. Buitrago, A. Sánchez, E. San Miguel, R. Villalba);; Centro de Investigación en Sanidad Animal, Valdeolmos, Spain (J. Fernández-Pinero, M. Elizalde, F. Llorente, M.A. Jiménez-Clavero)

**Keywords:** Bagaza virus, flavivirus, viruses, wild birds, partridges, pheasants, nucleotide sequences, Spain, dispatch

## Abstract

In September 2010, an unusually high number of wild birds (partridges and pheasants) died in Cádiz in southwestern Spain. Reverse transcription PCR and virus isolation detected flavivirus infections. Complete nucleotide sequence analysis identified Bagaza virus, a flavivirus with a known distribution that includes sub-Saharan Africa and India, as the causative agent.

An essential feature of certain emerging pathogens is their ability to expand their geographic ranges. Several arboviral diseases, particularly those caused by flaviviruses, have been found in new areas beyond their usual ranges. The best example of this phenomenon was introduction of West Nile virus into the Americas in 1999 (*1*). Other recent expansions of flaviviruses were the introductions of Japanese encephalitis virus into Australia in 1995–1998 (*2*) and Usutu virus into Europe in 2001 (*3*).

We report an outbreak of disease in wild birds (partridges and pheasants) in Spain that was caused by a flavivirus, Bagaza virus (BAGV). This virus was first isolated in Bagaza, Central African Republic, in 1966, from a pool of mixed-species female *Culex* spp. mosquitoes (*4*). It has subsequently been found in mosquitoes in other countries in western Africa (*5**,**6*) and in India, where serologic evidence suggests that this virus may infect humans (*7*), although its pathogenicity in humans is uncertain. BAGV has been shown to be synonymous with Israel turkey meningoencephalitis virus, a pathogen affecting poultry (turkeys) and reported only in Israel and South Africa (*8*).

## The Study

In September, 2010, an unusually high number of red-legged partridges (*Alectoris rufa*) died on several hunting properties in southwestern Cádiz, the southernmost province in Andalusia, Spain. Clinical signs included weakness, prostration, lack of motor coordination, weight loss, and white diarrhea. Some common pheasants (*Phasianus colchicus*) were also affected. These findings coincided in time with the first cases of West Nile virus (WNV) infection in horses detected in Spain (*9*).

Because red-legged partridges have recently been shown to be susceptible to WNV disease (*10*), it immediately raised suspicions that WNV could be responsible for these deaths. Therefore, samples were obtained from dead partridges (n = 11) and pheasants (n = 2) and sent to the Spanish Central Veterinary Laboratory in Algete for diagnostic analysis. The carcass of 1 of the affected partridges was also subjected to necroscopic analysis of different tissues, including heart, intestine, lung, liver, kidney, brain, and feathers (Table).

**Table:** Analysis of red-legged partridges (*Alectoris rufa*) and common pheasants (*Phasianus colchicus*) for WNV and BAGV, Cádiz, Spain, 2010*

Species	No. animals analyzed	Sample type	No. positive/no. tested		Virus isolation in ECE, no. samples positive/no. tested†
			WNV	BAGV	
*A. rufa*	11	Brain	0/11	11/11	1/3 (AF)
		Cloacal swab	0/2	1/2	NA
		Oral swab	0/2	1/2	NA
		Gut	0/1	1/1	NA
		Heart‡	0/1	1/1	1/3 (AF)
		Kidney‡	0/1	1/1	2/3 (AF, CM, and VS)
		Lung	0/1	1/1	NA
		Liver	0/1	1/1	NA
		Blood	0/1	1/1	NA
		Feathers§	0/3	3/3	NA
*P. colchicus*	2	Brain	0/2	2/2	NA

*West Nile virus (WNV) or Bagaza virus (BAGV) nucleic acids were identified by using real-time reverse transcription PCR (RT-PCR) or panflaviviral heminested RT-PCR, respectively, and amplicon (214 bp) sequencing. ECE, embryonated chicken egg; AF, allantoic fluid; NA, not analyzed; CM, chorioallantoic fluid; VS, viscera. †ECEs were infected with homogenates of various samples. ‡Samples from which full-length sequence of BAGV was obtained. §Tail, breast, and wing feathers from 1 partridge were analyzed.

Analysis by real-time reverse transcription PCR (RT-PCR) specific for lineage 1 and lineage 2 WNV (*11*) showed negative results for all samples tested. However, a heminested RT-PCR specific for a broad range of flaviviruses (*12*) showed positive results for all samples (Table), indicating that a non-WNV flavivirus was present in samples from different tissues of diseased birds.

cDNA obtained after real-time RT-PCR (252-bp fragment) or heminested RT-PCR (214-bp fragment) amplification of samples was subjected to nucleotide sequencing. Resulting nucleotide sequences were compared with those in GenBank by using BLAST analysis (www.ncbi.nlm.nih.gov). Our isolates had >90% homology with 2 BAGV strains in GenBank (strain DakAr B209 from Africa, GenBank accession no. AY632545, and strain 96363 from India, GenBank accession no. EU684972).

Virus isolation was performed by infection of embyonated chicken eggs with tissue homogenates from an infected partridge and confirmed by pan-flaviviral RT-PCR and nucleotide sequencing (Table). Virus was detected more frequently in allantoic fluid of infected eggs than in other egg tissues (Table). Further propagation was accomplished in BSR cells, a clone of baby hamster kidney-21 cells.

Genomic characterization of virus was performed by bidirectional sequencing of real-time RT-PCR fragments amplified from heart and brain samples from 1 of the affected partridges. We used 27 primer sets designed for this study on the basis of sequences for BAGV in GenBank. Full-length genome sequences were obtained by assembling overlapping nucleotide sequences and using the SeqScape program (Applied Biosystems, Foster City, CA, USA). Two full-length genome sequences obtained from heart (BAGV Spain H/2010, GenBank accession no. HQ644143) and brain (BAGV Spain B/2010, accession no. HQ644144), were identical.

**Figure 1:**
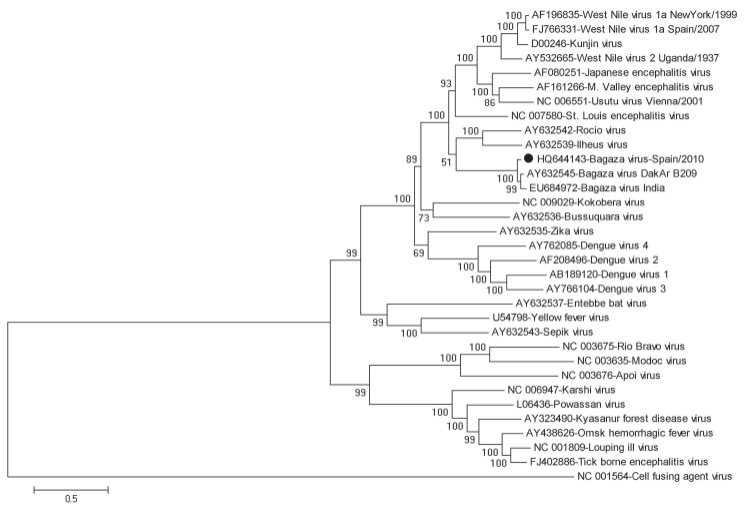
Phylogenetic relationships between a full-length genomic sequence for Bagaza virus identified in Cádiz, Spain, 2010 (solid circle) and 32 full-length flavivirus sequences, including 2 Bagaza virus isolates from GenBank. The phylogenetic tree was inferred by using the maximum-likelihood method. Percentage of 500 successful bootstrap replicates is indicated at the nodes. Evolutionary distances were computed by using the optimal general time reversible + Γ + proportion invariant model. A discrete Γ distribution was used to model evolutionary rate differences among sites (5 categories, G parameter = 2.0552). The rate variation model enabled some sites to be evolutionarily invariable (+I, 10.1524% sites). The tree is drawn to scale, and branch lengths are indicated as number of nucleotide substitutions per site. There were 9,803 positions in the final dataset. Phylogenetic analyses were conducted by using MEGA5 (www.megasoftware.net). GenBank accession numbers are indicated beside each isolate/strain name.

**Figure 2:**
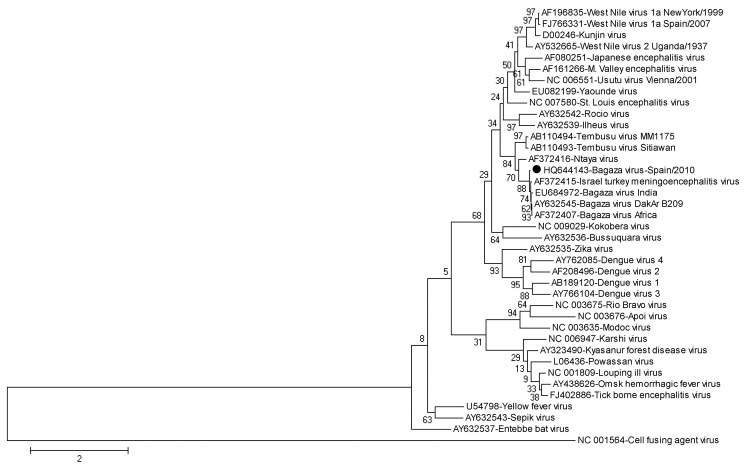
Phylogenetic relationships between partial envelope protein–coding gene sequence for Bagaza virus identified in Cádiz, Spain, 2010 (solid circle) and 38 equivalent flavivirus nucleotide sequences, including those for 3 Bagaza virus isolates, 1 Israel turkey meningoencephalitis virus, 1 Ntaya virus, and 2 Tembusu viruses from GenBank. The phylogenetic tree was inferred by using the maximum-likelihood method. Percentage of 500 successful bootstrap replicates is indicated at the nodes. The optimal Tamura-Nei with Γ distribution model was selected to compute evolutionary distances. A discrete Γ distribution was used to model evolutionary rate differences among sites (5 categories, G parameter = 1.8944). The tree is drawn to scale, and branch lengths are indicated as number of nucleotide substitutions per site. There were 941 positions in the final dataset. Phylogenetic analyses were conducted by using MEGA5 (www.megasoftware.net). GenBank accession numbers are indicated beside each isolate/strain name.

To assess phylogenetic relationships between these new BAGVs and other flaviviruses, the complete BAGV sequence obtained in this study (accession no. HQ644143) or a partial envelope (E) protein–coding gene subregion were aligned with other complete flaviviral genomes in GenBank by using the ClustalW algorithm in MEGA5 (*13*). Phylogenetic trees were constructed by using the complete BAGV genomic sequence or the partial E region (the E region is a well-known variable region in flaviviruses, and additional relevant nucleotide sequences are available in GenBank). Maximum-likelihood, neighbor-joining, or maximum-parsimony algorithms were used, and bootstrap tests with 500 replicates were performed to support each tree grouping. All generated trees showed similar topology and clustered the genomic sequence obtained in this study within the BAGV branch. Maximum likelihood trees are shown in Figures 1 and 2.

Sequence of BAGV from Spain was closely related to the 2 unique full-length BAGV sequences available in GenBank and showed greater similarity with the strain from Africa (94.1% nt identity) than with the strain from India (92.8% nt identity). Identity between the isolates from Africa and India was 95.0%, indicating that they are more related to each other than to BAGV from Spain. A total of 742 nt and 637 nt differences (70 nt and 47 aa differences) were observed between BAGV from Spain and the isolates from India and Africa, respectively. Genetic relatedness between all 3 viruses was high (>92%), which indicates that they belong to the same *Flavivirus* species (*Bagaza virus*). The tree based on the E region grouped Israel turkey meningoencephalitis virus and BAGV within the same cluster and showed that both viruses are closely related to Ntaya virus (Figure 2).

## Conclusions

BAGV has been detected and isolated in Cádiz, Spain. It appeared to seriously affect partridges and, to a lesser extent, pheasants, and caused an unusually high number of deaths in these birds. No signs of infection and no deaths were observed for other bird species. However, whether other bird species are susceptible to disease caused by BAGV should be determined because this virus is similar to Israel turkey meningoencephalitis virus, a relevant pathogen for turkeys. Also, other vertebrates could be at risk for infection with this virus. Thus, experimental studies on the pathogenicity of this virus in specific vertebrates should be conducted.

Transcontinental spread of flaviviruses has been often associated with bird migrations (*14**,**15*). Thus, infected birds migrating between Africa and Europe could have introduced BAGV into Spain. However, there is no evidence of transmission of this virus by migratory birds, and alternative explanations (poultry industry or trading of exotic birds for commercial or hunting purposes) should not be overlooked.
